# Latent Tuberculosis in HIV positive, diagnosed by the *M. Tuberculosis *Specific Interferon-γ test

**DOI:** 10.1186/1465-9921-7-56

**Published:** 2006-04-01

**Authors:** Inger Brock, Morten Ruhwald, Bettina Lundgren, Henrik Westh, Lars R Mathiesen, Pernille Ravn

**Affiliations:** 1 Department for Infectious Diseases, University Hospital, Hvidovre 2650, Denmark; 2 Department for Clinical Microbiology, University Hospital, Hvidovre 2650, Denmark

## Abstract

**Background:**

Although tuberculosis (TB) is a minor problem in Denmark, severe and complicated cases occur in HIV positive. Since the new *M. tuberculosis *specific test for latent TB, the QuantiFERON-TB In-Tube test (QFT-IT) became available the patients in our clinic have been screened for the presence of latent TB using the QFT-IT test. We here report the results from the first patients screened.

**Methods:**

On a routine basis the QFT-IT test was performed and the results from 590 HIV positive individuals consecutively tested are presented here. CD4 cell count and TB risk-factors were recorded from patient files.

**Main findings:**

27/590(4.6%) of the individuals were QFT-IT test positive, indicating the presence of latent TB infection. Among QFT-IT positive patients, 78% had risk factors such as long-term residency in a TB high endemic area (OR:5.7), known TB exposure (OR:4.9) or previous TB disease (OR:4.9). The prevalence of latent TB in these groups were 13%, 16% and 19% respectively. There was a strong correlation between low CD4 T-cell count and a low mitogen response (P < 0.001;Spearman) and more patients with low CD4 cell count had indeterminate results.

**Conclusion:**

We found an overall prevalence of latent TB infection of 4.6% among the HIV positive individuals and a much higher prevalence of latent infection among those with a history of exposure (16%) and long term residency in a high endemic country (13%). The QFT-IT test may indeed be a useful test for HIV positive individuals, but in severely immunocompromised, the test may be impaired by T-cell anergy.

## Introduction

Tuberculosis (TB) is the most prevalent disease in human immunodeficiency virus (HIV) positive and the majority of the people at risk of HIV and TB are living in Sub Sahara Africa [1]. The risk of developing active TB in HIV positive individuals is increased many fold even when antiretroviral chemotherapy is given [2,3] and the incidence of TB is increasing in regions where HIV is prevalent [1]. To prevent further spread of TB, intensified efforts are needed such as active case finding, and reducing the risk of reactivating TB in persons with latent tuberculosis infection (LTBI), through prophylactic and antiretroviral treatment.

Diagnosis of early sputum negative tuberculosis and LTBI has been hampered by low performance of the tools currently available. The Tuberculin Skin Test (TST) based on Purified Protein Derivative (PPD) has been the most important tool for the detection of LTBI for almost a century. The main drawback of the TST is the lack of specificity due to cross reactivity with proteins present in other mycobacteria such as the Bacille Calmette Guèrin (BCG) vaccine strain, *M. avium *complex organisms, and other non tuberculous mycobacteria [4-7]. In addition, the sensitivity of the TST is reduced in HIV positive patients[2,4,8].

Identification of 3 *M. tuberculosis *specific antigens, ESAT-6, CFP-10, and TB 7.7, has led to the development of a whole new generation of *M. tuberculosis *specific diagnostic tests [6]. ESAT-6, CFP-10, and TB 7.7 are contained within the regions of the mycobacterial genome which are absent from *M. bovis *BCG, *M. avium *and most other non-tuberculosis mycobacteria [6,9-11]. The *M. tuberculosis *specific tests are based on the stimulation of sensitized T-lymphocytes followed by measurement of interferon-γ (IFN-γ) by either enzyme linked immunoassay (ELISA) or enzyme linked immunospot assay. The latest improvement within this technology is the QuantiFERON^® ^TB-Gold In-Tube test (Cellestis, Australia), in which whole blood is drawn directly into vacutainer tubes precoated with antigens ready for incubation (QFT-IT test).

The sensitivity of the *M. tuberculosis *specific IFN-γ tests for the diagnosis of active TB has in most studies been >85% [12-15] and specificity in low TB risk populations 97–100% [1,5,7,12-17] with a low number of indeterminate test results. There is high a correlation between a positive antigen specific IFN-γ response and the degree of TB exposure [18-21] indicating that the test detects recent and latent infection. Cross reactivity has not been reported neither in healthy BCG vaccinated individuals [12,13,16,17,20,22] nor in patients infected with *M. avium*[5,7]. The only other mycobacteria which have been shown to induce T-cell recognition of ESAT-6 and CFP-10 are *M. marinum *and *M. kansasii*[23]. Only very limited information is available on the performance of IFN-γ tests in HIV positive and immunocompromised individuals.

The incidence of TB in Denmark is low <10/10^5 ^inhabitants/year [24] and the prevalence of HIV infection is 70/10^5 ^inhabitants[25]. Every year 10–12 HIV positive individuals are diagnosed with active TB[24], corresponding to a strikingly high incidence of TB in HIV positive of >300/10^5 ^individuals/year. This high incidence suggests a need for greater awareness and improved TB control measures among HIV positive. When the *M. tuberculosis *specific IFN-γ tests became commercially available in Denmark in 2004, we decided to screen our patients for LTBI using the QFT-IT test. We here report the results of the first 590 HIV positive individuals who were tested with the QFT-IT test.

## Materials and methods

### Study population

We used the QFT-IT test to screen for LTBI among the HIV positive patients attending the outpatient clinic at the University Hospital Hvidovre, Denmark. The clinic monitors 1300 HIV positive patients out of a total of approximately 3050 known HIV positive persons in Denmark[25]. Patients were screened during their routine quarterly check-up. This check-up comprises clinical evaluation, control of viral load, CD4-cell count, compliance with treatment, and an evaluation of potential side effects. Patients with a positive QFT-IT test were evaluated to rule out active TB. By reviewing the patient files, clinical information was recorded, such as age, sex, ethnicity, year of HIV diagnosis, AIDS defining diagnosis, previous treatment for active TB, and on tuberculosis risk factors such as exposure to an index case with sputum positive TB, history of long term residency in a high endemic country (defined as a country with a TB incidence >25:10^5^), intravenous drug use (IVDU) and factors assumed to influence immune status such as alcohol abuse or diabetes.

### QuantiFERON^®^TB-Gold In-Tube test

The QFT-IT test was performed at the Department for Clinical Microbiology according to the manufacturer's instructions; Briefly, 1 mL of blood was drawn directly into vacutainer tubes coated with saline (negative control), peptides of ESAT-6, CFP-10 and TB-7.7, or PHA (mitogen control). Tubes were incubated for 20 hours at 37°C, and plasma was harvested and frozen until further analysis. The amount of IFN-γ produced was determined using ELISA. IFN-γ release in the saline control tube (Nil) was subtracted from the TB antigen and PHA stimulated tubes. Samples with ≥0.35 IU/mL IFN-γ following stimulation with *M. tuberculosis *specific antigens were considered positive, while samples with <0.35 IU/mL were considered negative. The QFT-IT test result was considered indeterminate if production of IFN-γ after stimulation with PHA was < 0.5 IU/mL; indeterminate results could be due to technical errors or anergy. Calculations were performed using software provided.

### Statistical methods

Median values, 25^th ^and 75^th ^percentiles and mean ± standard deviations are shown. Median values were compared using Mann Whitney ranked sum test. Trend analysis was performed using non-parametric test for trend across ordered groups. Chi-square test and Mc Nemar tests were used to compare proportions. The correlation between CD4-cell count and the level of response to PHA was assessed using Spearman correlation test. Calculations were performed using SAS, Med Calc and the SISA software .

From the Danish Data Protection Agency, permission to analyze on data from the patient files was obtained (Jr.nr.2005-41-5520).

## Results

The screening was initiated October 2004 and by January 2005, 607 patients had been tested. In 17 patients, QFT-IT results were not available due to technical or logistic errors (i.e. samples left for too long before incubation, the patient failed to go to the laboratory for the test) and were not included in the data analysis. Baseline information for 590 HIV patients are shown in Table 1. Based on data from the Danish HIV cohort[25], we believe that the patients included are representative for the entire Danish HIV population.

**Table 1:** Baseline data for all the HIV positive screened with the QFT in tube test.

	**All 590**
**Age**, median years (25th–75th quartile)	43 (37–50)
**Male **sex, no (%)	434 (74)
**Ethnicity, no (%)**	

Danish	440 (74)
Non-Danish European	26 (4)
North American	4 (1)
African	78 (13)
Asian	26 (5)
Middle Eastern	10 (2)
Latin American	7 (1)
**Tuberculosis **no. (%)	

Previous TB diagnosis	31 (5)
**Tuberculosis risk factors**, no (%)	
History of exposure,	60 (10)
Long term residence in a TB endemic country ^a^	122 (21)
IVDU	69 (12)
≥1 risk factor	218(37)
**HIV status**	

Age at HIV diagnosis, median years (25th–75th quartile)	34 (28–41)
Years with HIV diagnosis, median years (25th–75th quartile)	8.5 (4–14)
AIDS diagnosed, no (%)	118 (20)
HAART treatment, no (%)	448 (76)
**CD4 cell count**	
CD4 cell count, mean (± s.d.)	523 (± 278)
0–99 CD4 cells/μL, (%)	17 (3)
100–199 CD4 cells/μL, (%)	37 (6)
200–300 CD4 cells/μL, (%)	63 (11)
>300 CD4 cells/μL	473 (80)
**HIV RNA, no (%)**	

<500 copies/mL	439 (74)
500 – 50,000 copies/mL	95 (16)
>50,000 copies/mL	56 (9)
**Factors presumed to influence immune status**	

Diabetes, no. (%)	20 (3)
Alc. Abuse, no (%)	63 (11)

^a^Incidence of TB >25/100,000.

### Baseline IFN-γ responses

**Figure 1:**
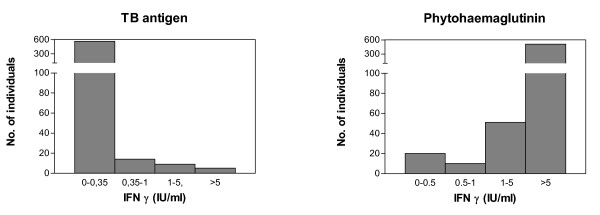
IFN-γ release after stimulation of whole blood with either TB antigen (left) or Phytohaematglutinin (PHA)(mitogen) (right). All results are stratified into intervals according to the level of IFN-γ released after stimulation and the number of individuals with IFN-γ release within each interval are shown.

**Figure 2:**
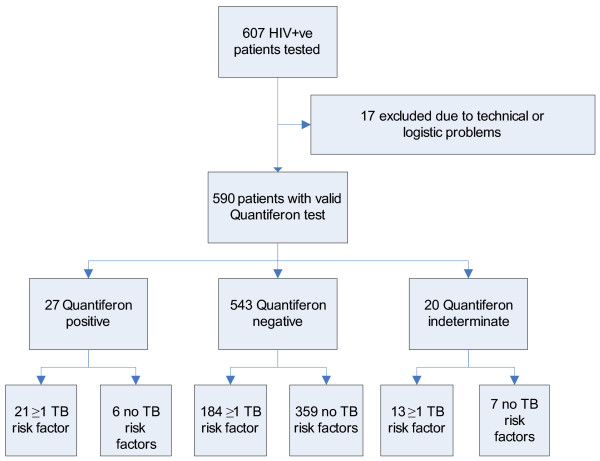
Study Flow Diagram for 607 HIV positive patients screened with the QFT- IT test. TB risk factors implies: prior TB diagnosis, history of TB exposure, history of long-term residency in a TB high endemic country (>25 cases per 100.000/year).

The distribution of IFN-γ released upon stimulation with either PHA or ESAT-6, CFP-10 and TB-7.7 are shown in Figure 1. During the first months, 33 patients had blood drawn twice with an interval of 1–12 weeks. Very low variation was seen between the two time points. The median IFN-γ production were 19.47 IU/mL and 19.74 IU/mL respectively after PHA stimulation and 0.01 IU/mL and 0.01 IU/mL after stimulation with antigen (data not shown). Three individuals changed from an indeterminate PHA response to a positive response or the opposite. Patient 1. changed from 1.72 IU/ml to 0.25 IU/ml, patient 2. from 1.69 IU/ml to 0,16 IU/ml, and patient 3. from 0.07 IU/ml to 6,72. Only patient converted from a negative (0.22 IU/ml) to a positive QFT-IT response (0.42 IU/mL) in the second test 3 month later. This patient had been visiting relatives in a high incidence area between the two tests. The remaining patients were QFT-IT test negative on both tests. For data analysis the first sample was always used. Of the 590 patients screened, 20(3.4%) patients were not able to mount an IFN-γ response above the cut-off (>0.5 IU/mL) in response to PHA and their QFT-IT test results were considered "indeterminate". Of 570 patients with determinate test results, 27 were QFT-IT test positive and 543 were QFT-IT test negative (Figure 2).

### Prevalence latent and incidence of active TB

The overall prevalence of LTBI, determined by a positive QFT-IT test result, was 4.6% (27/590) and 4.7% (27/570) when patients with an indeterminate response were excluded. After excluding all patients with a history of previous TB or indeterminate QFT-IT test result, the prevalence was 4.1% (22/542). One patient was already on chemotherapy when the QFT-IT test was done and all patients with positive QFT-IT tests were screened for active TB. Within A one year period from of the QFT-IT test was performed 2 patients, both QFT-IT positive, were diagnosed with active TB. One of the patients had completed 9 months of treatment 1 year prior, but had a relapse of pulmonary TB. The other patient had been intensively investigated for active TB due to unspecific symptoms and 9 month after the QFT-IT was done active TB was and finally diagnosed in an abdominal lymph gland. Despite the low number of TB cases, the incidence of active TB within the year of the screening was extremely high 7.407:10^5^(2/27).

### Identification of risk factors

Risk factor correlation was determined using results from the 570 individuals with a valid QFT-IT test result (PHA response >0.5 IU/mL). Covariates including age, sex, year of HIV diagnosis, AIDS diagnosis, CD4-count, HIV RNA, prior TB diagnosis, factors presumed to influence immune status (alcohol abuse or diabetes) and tuberculosis risk factors (history of TB exposure, long term residency in a high endemic country and IVDU) were analysed (Table 2). The patients with a positive QFT-IT test had significantly more risk factors than the QFT-IT negative patients; 78% (21/27) of the QFT-IT positive patients had one or more TB risk factors in contrast to 34% (184/543) of the patients with negative QFT-IT test (OR 7.2, CI: 2.8–18.2, p= 0.00003). Of those with an indeterminate test, 65%(13/20) had risk factors for TB. Despite the low number of patients with a positive QFT-IT test result, we identified groups of individuals with an increased risk of LTBI: patients with a history of TB exposure (OR 4.9, CI: 2.0–11.8, p= 0.001154) and patients with long term residency in a high endemic country (OR: 5.7, CI: 2.6–12.5, p = 0.00002). Among patients with a history of previously treated TB, there was an increased risk of a positive QFT-IT test(OR: 4.9, CI: 1.7–14.1, p= 0.007). In contrast, age, IVDU, alcohol abuse or diabetes, CD4-cell count or viral load were not associated with positive QFT-IT test results. The calculated odds ratios translated into a prevalence of LTBI among HIV positive patients with a long term residency in a high endemic country of 13% (15/123) among patients with a history of exposure of 16% (8/51), and among patients with previous TB of 19% (5/27).

**Table 2:** Identification of risk factors for LTBI among HIV positive

	QTF negative (n = 543)	QTF positive (n = 27)	OR, (95% CI)	P values
Age, median years (25th–75th quartile)	43 (38–50)	42 (36–49)		P = 0.05
Male sex, no (%)	405 (75)	17 (63)		
Tuberculosis				
Previous TB diagnosis no. (%)	22 (4)	5 (19)	4.9 (1.7–14.1)	p= 0.0063
Tuberculosis risk factors, no. (%)				
History of exposure	43 (8)	8 (30)	4.9 (2.0–11.8)	p= 0.001154
Long term residence in a TB endemic country ^a^	98 (17)	15 (56)	5.7 (2.6–12.5)	p= 0.000021
IVDU ^b^	59 (11)	5 (19)		P= 0.110
≥1 risk factor	184 (34)	21 (78)	7.2 (2.9–18.2)	p= 0.000003
HIV status				
Age at HIV diagnosis, median years (25th–75th quartile)	34 (28–41)	34 (29–40)		P = 0.11
Years with HIV diagnosis, median years (25th–75th quartile)	8.5 (4–14)	7 (4–11.0)		P = 0.10
AIDS diagnosed, no (%)	107 (20)	6 (22)		P = 0.18
HAART treatment, no (%)	416 (77)	19 (70)		P = 0.13
CD4 cell count, mean (± s.d.)	523 (± 273)	600 (± 274)		P = 0.17
0–99 CD4 cells/μL, (%)	13 (2)	0 (0)		
100–199 CD4 cells/μL, (%)	35 (6)	1 (4)		
200–300 CD4 cells/μL, (%)	55 (10)	2 (7)		
>300 CD4 cells/μL,l	440 (81)	24 (89)		
HIV RNA, no (%)				
<500 copies per ml	412 (76)	18 (67)		0.10
500 – 50,000 copies per ml	79 (15)	8 (30)		
>50,000 copies per ml	52 (10)	1 (4)		
Factors presumed to influence immune status				
Diabetes, no. (%)	18 (3)	2 (7)		0.18
Alc. Abuse, no (%)	59 (11)	3 (11)		0.24

^a^Incidence >25/100,000.

^b^Intravenous drug user

Differences between media n values were analysed using Mann-Withney test, and differences between proportions using Chi Square test

Nine-teen percent (5/27) of the QFT-IT positive individuals were previously treated for TB. The median interval between TB diagnosis and the present QFT-IT test was 1,5 years (25^th^–75^th ^quartile 0.5–4.3 years) for the patients with a positive QFT-IT test in contrast to 6.5 years (25^th^–75^th ^quartile 4.4–9.4 years) for patients with a negative QFT-IT test (p = 0.019, data not shown).

### Influence of low CD4 cell count on the outcome of the QFT-IT test

**Figure 3:**
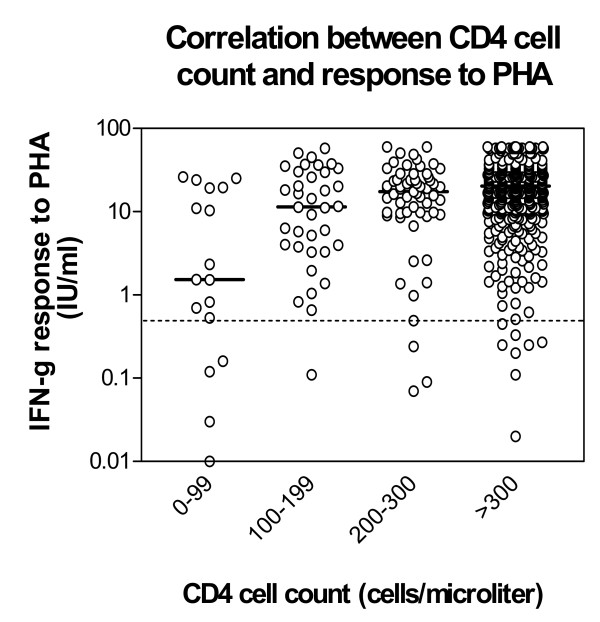
IFN-γ release (IU/mL) after stimulation of whole blood with PHA (mitogen). Results are stratified into intervals according to the CD4-cell count for each individual. The median IFN-γ values within each CD4-cell count interval are indicated with horizontal lines. Cut off for PHA response of 0.5 IU/ml is shown by a dotted line.

We analyzed the possible effect of a low CD4 count on the test performance and found a strong correlation (p < 0.001 Spearman) between the CD4-cell count and the level of PHA stimulated IFN-γ production (data not shown). The level of IFN-γ produced in response to PHA by each individual has been stratified according to the level of CD-4 cell count and is shown in Fig. 3. The median IFN-γ levels as well as the proportion of patients with indeterminate results is shown in Table 3. The median IFN-γ release in the group with a CD4-cell count <100 cells/μL was significantly lower than any of the three other groups (Mann-Whitney ranked sum test: p < 0.0001) and there was a trend for increasing INF-γ release for increasing CD4 group (non-parametric test for trend across ordered groups (p < 0.0001). In addition, 24% (4/17) of the patients with a CD4-cell count <100 cells/μL had an indeterminate test result due to low INF-γ production following PHA stimulation compared to only 2.8%(16/573) of the patients with a CD4 cell count >100 cells/μL (p < 0.0005).

**Table 3:** Association between CD4 cell count and IFN-γ release in response to PHA.

**CD4 cell counts**^**1)**^	**No of patients**	**Number and percentage of PHA non-responders**	**IFN-γ release (Median and 25–75 percentile)**
<100	17	4 (24%)*	1,53 (0,34-19,49)^#^
100–199	37	1 (3%)	11.58 (3,88-30,11)
200–299	63	5 (8%)	16,78 (9,40-25,20)
>300	473	10 (2%)	20,35 (13,20–31,00)

1) cells/μl

* p < 0.0005, difference in the proportion of PHA-non-responders between patients with a CD4 cell count higher or lower than 100 cells/μL (Chi-Square test).

# p = 0.0001 difference in the median IFN-γ release between patients with a CD4 cell count < 100 compared to groups of patients with a CD4 cell count ≥ 100 cells/μL (Mann-Withney U test) and there was a trend for increasing INF-γ release for increasing CD4 group (non-parametric test for trend across ordered groups (p < 0.0001).

## Discussion

We found that the overall prevalence of LTBI (defined by a positive QFT-IT test) of 4.6% among HIV positive in Denmark was relatively low, but the prevalence of LTBI was much higher among those with a history of residence in a TB high endemic country (13%) or with known TB exposure (16%). In low endemic countries very low prevalence of LTBI (0–3%) has been found independently of BCG vaccination status [12,13,15-17,20]. In an intermediate endemic burden country, the prevalence of LTBI in the general population was low 4%, increasing to 21% in contacts of sputum positive TB patients[18]. In TB high endemic regions, the prevalence of LTBI in the population was 30%[19] increasing to 60–70% in household contacts of TB patients[16,19] and to 40% in a group of Indian health care workers[26]. Together, the QFT-IT test appears to be very efficient in specific identification of individuals at risk of LTBI. We did not find an association between IVDU or alcohol abuse and being QFT-IT test positive. This was unexpected since the impression is that there is an increased risk of TB among patients with IVDU and alcoholics in Denmark due to increased risk of exposure[24,27].

Patients with a history of previously treated TB had a high rate of positive QFT-IT test results(19%). The rate was highest in patients with recent TB compared to patients with TB many years ago suggesting that the QFT-IT test response may diminish over time. There is an ongoing debate whether it is possible to monitor the effect of chemotherapy treatment using INF-γ based tests[28,29], but the present study and previous reports[13,30], indicate *M. tuberculosis*-specific *responses *can be retained for many years after treatment. Whether this reflects differences in long term memory immunity or insufficient treatment leading to persistent or latent infection is unknown.

There is a relevant concern that the performance of the QFT-IT test, in line with the TST, could be impaired by low sensitivity in patients with advanced immunodeficiency. The PHA control serves as a surrogate marker for anergy as well as a quality control of the assay. In patients with a CD4-cell count <100 cells/μL, we found a higher proportion of indeterminate test results (24% vs 2.8%) due to low PHA response and there was a significant correlation between CD4-cell count and PHA induced IFN-γ release. These findings support the hypothesis that the performance of the IFN-γ based tests is impaired in patients with advanced immunosuppression. *In vivo and in vitro *anergy in HIV positive individuals is well recognized [2-4,8,31,32] and the reduced antigen response correlates proportionally with CD4-cell count and is reversible during antiretroviral treatment[3,32]. Fisk and colleagues found that a CD4-cell count <100 cells/μL, was the critical level associated with skin test anergy[32]. Limited data are available on the performance of the IFN-γ test in HIV positive individuals. Converse and colleagues found, using the 1^st ^generation QuantiFERON^® ^test based on tuberculin PPD, a reduced rate of responders and a lower mean IFN-γ response in HIV positive individuals with a low CD4-cell count (<200 cells/μL)[8]. Other studies [15,33] have used the ELISPOT test based on ESAT-6 and CFP-10 and found that the number of responders as well as the mean IFN-γ response was reduced in HIV positive individuals. However neither CD4-cell count or AIDS diagnosis, were available in either study. A small study including 29 HIV positive patients surprisingly concluded that there was no impact of a low CD4-cell count on the performance of the IFN-γ response in an ELISPOT assay[34]. However, based on our findings and the numerous reports demonstrating reduced T-cell function in HIV patients it is most likely that the *M. tuberculosis *specific IFN-γ responses are impaired in patients with advanced immunosuppression in both ELISPOT and whole blood assays.

There is not yet consensus on how to determine the critical level of immunosuppression which may impair the performance of the tests. Reducing the cut off level is a possibility, but may result in the loss of specificity. An alternative is to define a cut-off level of CD4-cells at which the sensitivity of the IFN-γ is impaired. Until further information has been obtained, we argue that PHA is used as the currently best described control and emphasize the need for careful interpretation of an indeterminate or negative test result in HIV positive with low a CD4 cell count (i.e. <100–200 cells/μL). Despite these considerations it is worth noticing that 76% of the patients with a low CD4-cell count did respond to stimulation with PHA.

TST was not performed in parallel with the QFT-IT screening, which is of course a constrain to the overall evaluation of the results. TST however, is not routinely performed in our patients because most of the HIV positive patients were born before 1975 and thus BCG vaccinated and a poor specificity of the TST is expected. Despite this, a TST would have contributed to understanding of the differences between the TST and QFT-IT in HIV positive.

### Perspectives

The *M. tuberculosis *specific QFT-IT test offers methodological and logistic advantages over the TST. It requires only one patient visit and plasma can be frozen for later analysis. It does not induce a boosting phenomenon that is seen with the TST due to repeated injections of mycobaterial antigens. Once established in the laboratory, reproducibility is high as shown herein. The number of steps resulting in direct contact with potentially contagious blood from HIV positive individuals is minimal with the In-Tube test system, thus reducing the occupational hazards of performing TB testing. By introducing the *M. tuberculosis *specific tests, we may be able to improve TB control by specific identification of those HIV positive individuals with LTBI. A positive QFT-IT test is strongly suggestive of LTBI whereas an indeterminate test result or a negative QFT-IT test result in the severely immunocompromised hosts should always be interpreted with caution

## Conclusion

Using the QFT-IT test, we found an overall prevalence of LTBI of 4.6% in HIV positive individuals and a much higher prevalence of LTBI among patients with known exposure(16%) and residency in a high endemic country(13%). Two cases of active TB was found among 27 QFT-IT positive patients resulting in an extremely high incidence of TB (7.4%). The QFT-IT test may be a useful new test for detecting LTBI in immunocompetent HIV positive individuals and future studies should be designed to determine the critical lower level of CD4-cells and to determine the role of these tests in high endemic regions.

## Competing interests

The author(s) declare that they have no competing interests.

## Authors' contributions

Inger Brock and Morten Ruhwald have contributed equally to the study.

## Financial support

has been obtained by Danish AIDS foundation

Hvidovre Hospital Research Foundation.

Reagents have been supplied at reduced price by Cellestis Ltd. Australia and Statens Serum Institute, Denmark.
